# The Use of Synthesized CoO/Co_3_O_4_ Nanoparticles as A Corrosion Inhibitor of Low-Carbon Steel in 1 M HCl

**DOI:** 10.3390/ma15093129

**Published:** 2022-04-26

**Authors:** Ghadah M. Al-Senani, Sameerah I. Al-Saeedi

**Affiliations:** Department of Chemistry, College of Science, Princess Nourah bint Abdulrahman University, P.O. Box 84428, Riyadh 11671, Saudi Arabia; sialsaeedi@pnu.edu.sa

**Keywords:** CoO/Co_3_O_4_ nanoparticles, corrosion, green synthesis, low-carbon steel, inhibitor, HCl

## Abstract

CoO/Co_3_O_4_ nanoparticles (CoO/Co_3_O_4_ NPs) were synthesized with egg white. The effectiveness of CoO/Co_3_O_4_ NPs to inhibit the corrosion of carbon steel has verified in acidic medium (1 M HCl). It has been found that Langmuir adsorption isotherm is the dominant adsorption process of CoO/Co_3_O_4_ NPs on the surface of low-carbon steel. The thermodynamic parameters also demonstrated that the adsorption process of CoO/Co_3_O_4_ NPs was a physicochemical, spontaneous, and exothermic process. The electrochemical impedance spectroscopy technique and potentiodynamic polarization were applied. The results obtained in this study showed that CoO/Co_3_O_4_ NPs acted as a mixed inhibitor for the anodic reaction and the cathodic reaction, and the efficiency to inhibit the corrosion was 93% at 80 ppm of the inhibitor. The results of scanning electron microscopy (SEM) technique, energy-dispersive X-ray spectroscopy (EDS), and X-ray electron spectroscopy (XPS) confirmed the effectiveness that was obtained using the inhibitor to protect the surface of low carbon steel. Thus, low-carbon steel can be protected against corrosion in acidic medium using CoO/Co_3_O_4_ NPs as inhibitors.

## 1. Introduction

It is well known that steel is divided into three types depending on the amount of carbon in the metal: low-carbon steel (carbon < 0.3%), medium-carbon steel (carbon from 0.3 to 0.6%), and high-carbon steel (carbon > 0.6%). Low-carbon steel contains a large amount of iron (98% to 99%). Low-carbon steel is often chosen because it is available in most countries, easy to install and weld, so it is used for tasks requiring strength and shaping [1]. It has enough carbon to make it hard and durable, but not brittle.

Therefore, companies prefer to use it in multiple phases (oil-water-gas) to ensure the continuation of the good life for a long time, as many pipelines connect to a single separation facility. This productive technology enables the pipeline to carry oil over a long distance and allows for an easy pipeline change, thus not affecting oil production [2,3]. Low-carbon steel is also used as a basic material for building chemical reactors, and in many industries, such as construction and transportation for its ability to withstand variable temperatures. However, low-carbon steel corrodes spontaneously in the environment due to chemical and electrochemical reactions, especially in acidic solutions [4,5,6].

A hydrochloric acid is the most prevalent stimulation. It corrodes all types of steel and most other alloys. In addition, hydrochloric acid is not an oxidizing agent, therefore, it does not cause passivation on the surface of alloy steels. The concentration of hydrochloric acid is 1M in general practice, since steel is highly reactive with hydrochloric acid, and higher concentrations may cause complete dissolution of the metal. The hydrochloric acid solution (1 M) is used for descaling, acid pickling operations, including hydraulic fracturing, to improve well productivity [7,8], and is used in many factories, petrochemical processes, acidification of oil wells, and to treat rust deposited in metalworking. During the pickling process and acid cleaning, pipe corrosion occurs that cannot and cannot be eliminated, prompting researchers to study the use of organic, inorganic, and natural corrosion inhibitors [9,10].

More recently, the focus has been on studying the incorporation of natural substances containing organic compounds with inorganic nanoparticles, such as Al_2_O_3_, ZrO_2_, SiO_2_, TiO_2_, CdS, NiO, ZnO, cobalt complex, and Fe_3_O_4_ [11,12,13]. This focus is due to nanoparticles’ mechanical and physical properties, which stem from their large surface area and small particle size [14].

The adsorption of nanoparticles at the surface of low-carbon steel could be both cathodic and anodic protection or one of them, and they provide protection in two basic ways. Chemical adsorption which includes transfer or share of charge between the inhibitor particle and charged metal surface, and physical adsorption consists of van der Waals interactions and the electrostatic attraction between charged inhibitors and the charged metal surface [15].

Cobalt oxides CoO and Co_3_O_4_ have a cubic crystal structure, in which Co_3_O_4_ crystallizes into a structure with the magnetic Co^2+^ ions at the tetrahedral sites and the non-magnetic Co^3+^ ions at the octahedral sites [16,17,18]. Despite the crystal similarity between Co_3_O_4_ and Fe_3_O_4_, Co_3_O_4_ exhibits a different magnetic order, different from the ferromagnetic ordering in Fe_3_O_4_. Cobalt oxides are also characterized by mechanical hardness, high electrical resistance, and chemical stability. These physical and chemical properties promote the formation of stable oxides on the surface of metals and alloys that contribute to the resistance to corrosion of low-carbon steels in acids, due to the formation of stable and continuous films through the interaction of Co with Fe and O [19,20].

Several chemical techniques, such as solubility-controlled synthesis, coprecipitation, combustion synthesis, freeze-drying, sol-gel, and spray-pyrolysis has been used to produce CoO and Co_3_O_4_ [16].

Limited studies have been found about the synthesis of cobalt oxides using egg whites as fuel during the preparation of the oxide.

In addition, there is little research on the effect of CoO/Co_3_O_4_ nanoparticles synthesized using egg white on the corrosion inhibition of low-carbon steel in acidic solution.

This research is a continuation of the previous work [21] and the aim of this research is to study the effect of CoO/Co_3_O_4_ nanoparticles synthesized using egg white on the inhibition of low low-carbon steel corrosion in 1 M HCl by electrochemical methods, and the application of kinetic, adsorption, and thermodynamic models to determine the adsorption mechanism of CoO/Co_3_O_4_ NPs.

## 2. Experimental Setup

### 2.1. Materials

All chemicals used in this study were of analytical grade. HCl—37%, and Co(NO_3_)_2_-6H_2_O—97%—LR, were purchased from Sigma-Aldrich Company Ltd. (St. Louis, MI, USA).

### 2.2. Synthesis of CoO/Co_3_O_4_ NPs

Cobalt oxides nanoparticles were synthesized using egg white extract in the same way in research [21], where cobalt nitrate was mixed with egg white extract at 80 °C with continuous stirring. After gel formation, roasting was completed at 350 °C, and a black powder containing cobalt oxides nanoparticles was obtained, which was detected by scanning electron microanalyzer (SEM) on a JEOL, JED-2200 Series (JEOL, Tokyo, Japan), transmittance electron microanalyzer (TEM) on a JEOL, JEM 2100 HRT (JEOL, Tokyo, Japan), energy dispersive spectroscopy (EDS), X-ray diffraction (XRD), and Fourier-transform infrared spectrum (FTIR) [21].

### 2.3. Solution Preparation

HCl (37%) were diluted using distilled water to prepare a stock solution of 1 M HCl. Next; the CoO/Co_3_O_4_ NPs powder (50 mg) were mixed with 1 M HCl (100 mL). The corrosion experiments were conducted in the absence and presence of the inhibitor at concentrations of 20; 40; 60 and 80 ppm.

### 2.4. Low-Carbon Steel Specimen Preparation

Low-carbon steel (X60) samples were obtained from SABIC (Saudi Iron and Steel Company in Jubail City) and consisted of C: 0.143%, this percentage is less than 0.3%, and it also consisted of Mn: 0.897, Si: 0.007, S: 0.004, P: 0.008, Cr: 0.009, Cu: 0.003, V: 0.004, Ni: 0.011, Ti: 0.001, Al: 0.044, Nb: 0.011, Ca: 0.0003, N: 0.003, Mo: 0.001, Co: 0.005, Sn: 0.0001% wt., and a balance of Fe. The samples were cut into cylinders and embedded in Teflon, and the exposed area was 1 cm^2^. Additionally, the samples were polished with 800 to 1200 grit emery papers and then washed with distilled water and acetone.

### 2.5. Electrochemical Measurements

Electrochemical impedance spectroscopy (EIS) and potentiodynamic polarization measurements were taken using a Gill AC instrument (125 Station Road, Cark, Grange-Over-Sands, Cumbria, United Kingdom. LA11 7NY). Three electrodes, the working electrode (low-carbon steel electrode), counter electrode (graphite electrode), and reference electrode (calomel electrode), used for all measurements. The open-circuit potential measured for 30 min to attain a steady-state. The EIS measurement frequency ranged from 0.01 to 10,000 Hz. Potentiodynamic polarization was applied in a potential range of ±250 mV vs. Eoc at a scan rate of 0.2 mVs^−1^.

The low-carbon steel electrode was immersed in 1M HCl in the absence and presence of CoO/Co_3_O_4_ NPs (10, 20, 40, 60 and 80 ppm) at 298 and 333 K.

The experiment was repeated three times, and the electrode was re-immersed in the solution for 48 h to ensure the effectiveness of the inhibitor.

The experiment was repeated three times with an error of ±0.05.

The following equations were used to calculate the inhibition efficiency (*IE*_inh_ (%)):

**EIS:**

(1) $$IE_{\mathrm{inh}}\left(\%\right)=\frac{\left(R_{\mathrm{ct}\left(\mathrm{inh}\right)}-R_{\mathrm{ct}}\right)}{R_{\mathrm{ct}\left(\mathrm{inh}\right)}}\times 100$$

**PDP:**

(2) $$IE_{\mathrm{inh}}\left(\%\right)=\frac{\left(i_{\mathrm{corr}}-i_{\mathrm{corr}\left(\mathrm{inh}\right)}\right)}{i_{\mathrm{corr}}}\times 100$$

where *R*_ct_ and *R*_ct(inh)_ denote the charge transfer resistance in the absence and presence of the inhibitor, respectively, and *I*_corr_ and *I*_corr(inh)_ are the corrosion current densities in the absence and presence of the inhibitor, respectively; these values were calculated from the intersection of the cathodic and anodic Tafel slopes.

### 2.6. The Surface Morphology Study

Scanning electron microscopy (SEM) and energy dispersive spectroscopy (EDS) were conducted with a JSM-6380 LA instrument (JEOL, San Diego, CA, USA) at a high resolution of 3.0 nm and an accelerating voltage of 0.5–30 kV.

X-ray electron spectroscopy was performed to characterize the CS surface by XPS measurements. Measurements were made on SpectrumScan_Qualitative_AlKa.csv, with passing an energy of 20 keV, with a total energy resolution of 1 eV, and an AlKα x-ray source (hν = 1486.6 eV).

## 3. Results and Discussion

### 3.1. Characterization of CoO/Co_3_O_4_ NPs

**Figure 1:**
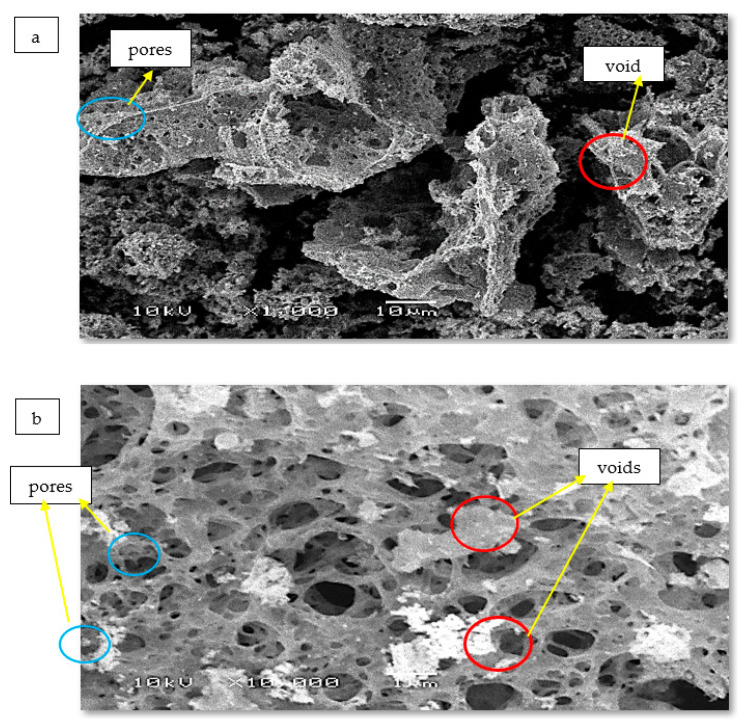

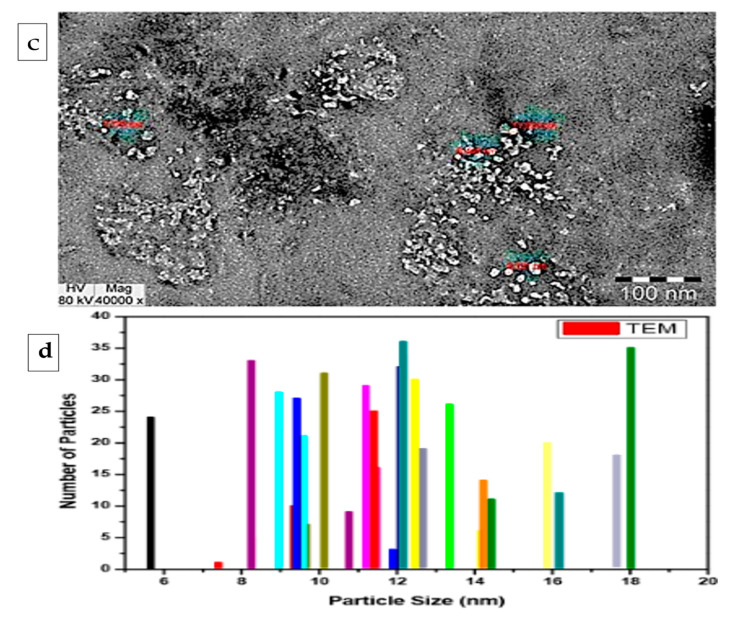
(**a**,**b**) SEM image of the as-prepared CoO/Co_3_O_4_ NPs with 1000× and 10,000× magnification, (**c**) TEM image of the as-prepared material, and (**d**) its histogram for particle size distribution.

SEM analysis showed the surface morphology of CoO/Co_3_O_4_ NPs (Figure 1a,b). Spongy clumps with pores and voids of different sizes are observed; high-contrast light nanoparticles, and cobalt oxides are seen clearly supported on a darker low-contrast porous material which is a carbon byproduct. These features are also clearly seen in low-magnification images. The particles clump together as spongy islands, which is why they do not appear as spherical as expected [21].

The analysis of the (TEM) pictures provides us with a clear morphologic characteristic of the synthesized sample as in Figure 1c. The morphology showed homogenous structures with sizes on the nanometer scale. Figure 1d showed the distribution of particles size histogram for the sample which supported that the partials were from 10–20 nm [21].

**Figure 2:**
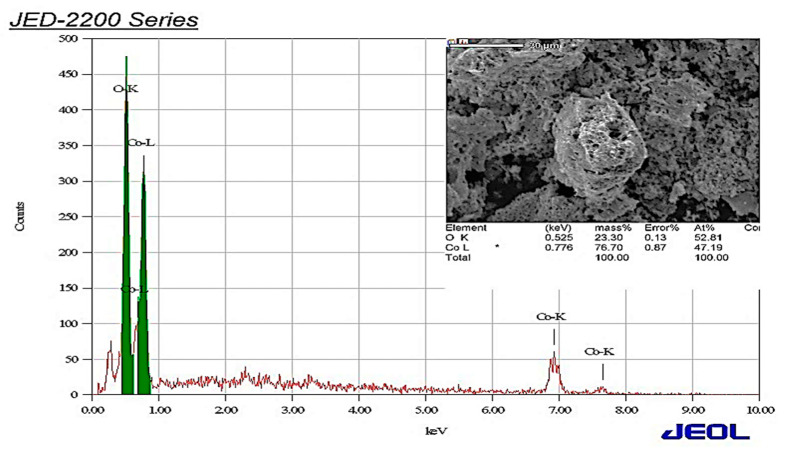
EDX pattern of the as-prepared CoO/Co_3_O_4_ NPs.

The CoO/Co_3_O_4_ NPs were accurately characterized in the previous study [21]. The EDX analysis was performed to confirm the presence of cobalt oxides, as it appears from the EDX spectrum that the main components of the prepared sample were Co and O elements. CoO/Co_3_O_4_ nanoparticles successfully synthesized (Figure 2) [21].

**Figure 3:**
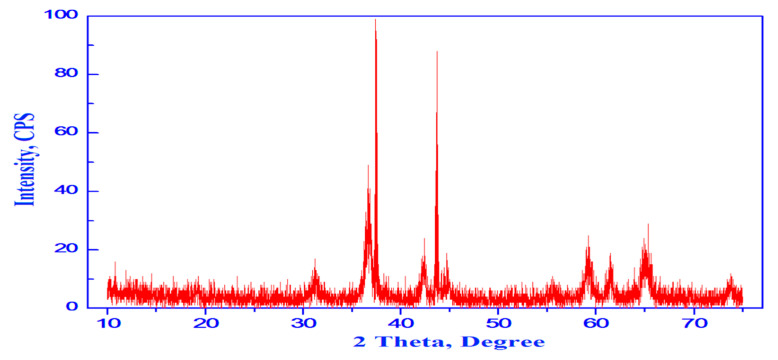
XRD pattern of CoO/Co_3_O_4_ nanoparticles.

Figure 3 Showed the XRD pattern of the synthesized CoO/Co_3_O_4_ NPs. A nanocomposite formation has been detected at the XRD trajectory, with the appearance of CoO and Co_3_O_4_. At 2θ degree a series of diffraction appeared as follows (31.19, 36.75, 38.25, 44.73, 55.47, 59.36, 65.37, 73.48, 76.67 and 77.7) which is characteristic to indexing planes (220), (311), (222), (400), (422), (511), (440), (620), (533) and (622), respectively; the values obtained in our study are in agreement with Co_3_O_4_ NPs. These diffraction peaks can be explained as typical cubic spline structure for Co_3_O_4_ (Major phase). In our study, other series of diffraction peaks at degree 2θ = 36.37, 42.24, 61.28, 73.39 and 77.24 characteristic to indexing planes (111), (200), (220), (311), and (222) were detected which in agreement with that reported for CoO NPs in other study [21]. Moreover, these diffraction peaks can be indexed as typical cubic structure for CoO NPs. In this study, no other phases were observed in the XRD patterns, and the carbonaceous byproduct appear as impurities due to the use of egg white in the preparation. XRD pattern indicate the formation of CoO/Co_3_O_4_ nanocomposite and most of the peaks present in the XRD belong to Co_3_O_4_ phase with fraction of cubic CoO.

**Figure 4:**
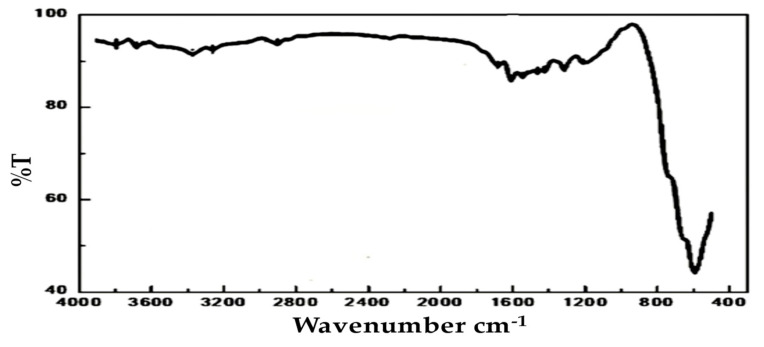
FTIR spectrum of CoO/Co_3_O_4_ nanoparticles.

Figure 4 show the spectrum FTIR of the sample. The result indicates the flowing (i) different bands at (3415, 1650, 1163, 643, 572, and 513 cm^−1^) with fundamental IR active modes in the vibration spectrum, with high frequency bands around 600 cm^−1^ at tetrahedral (A) site and low frequency bands around 400 cm^−1^ at octahedral (B) sites. It has been found in other study that the bands at 513 and 643 cm^−1^ spectrums are correlated with the vibration of Co^+3^ in octahedral and the Co^+2^ in tetrahedral hole in the spinel lattice [21]. The appearance of the shoulder band at 572 cm^−1^ is related to the divalent octahedral metal ion and oxygen ion complex and it could be related to the presence of Co^+2^ ions at B site.

### 3.2. Mechanism of Adsorption of Inhibitors on Low-Carbon Steel Surface

To guess the mechanism of adsorption of the inhibitor on the surface of the low-carbon steel, the corrosion mechanism of metal dissolution included in the hydrochloric acid solution, and it is known that the anode dissolution of low-carbon steel is [22,23]:

Fe + Cl^−^ → (FeCl)^+^_ads_ + 2e^−^ (3)

(FeCl)^+^_ads_ → Fe^2^^+^ + Cl^−^ (4)

The mechanism of the cathode reaction is the evolution of hydrogen:

Fe + H^+^ + e^−^ → (FeH)_ads_ (5)

(FeH)_ads_ + H^+^ + e^−^ → Fe + H_2_ (6)

Inhibition of low-carbon steel corrosion in HCl solution is conducted out by adsorption of inhibitor particles, which depend on the chemical components of the inhibitor, the surface nature of the low-carbon steel, the number of active sites and the temperature.

The physical and chemical adsorption process is carried out at the low-carbon steel/solution interface, by the following methods [4,24]:

1. The electrostatic attraction occurs between the charged metal and the charged damper particles.
2. A coordinated bond is formed between the free electron pairs in the inhibitor molecules and the vacant d-orbitals of the iron on the metal surface.
3. The electron donation and electron attraction reaction occur between the inhibitor particles and the metal.
4. All previous interactions.

Through the results obtained, it is clear that the low-carbon steel surface carries positive charges of Fe^2+^ ions that lead to the attraction and accumulation of Cl^-^ ions on the surface, which makes the surface negatively charged, which helps to attract and adsorb CoO/Co_3_O_4_ NPs onto the surface of low-carbon steel.

**Figure 5:**
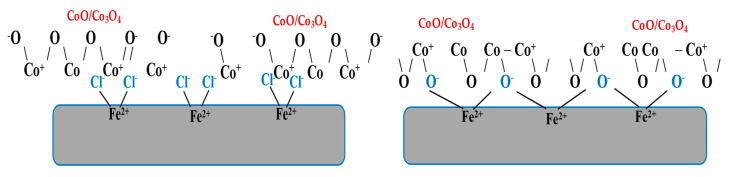
Mechanism of inhibiting low-carbon steel corrosion.

Furthermore, the presence of Co^2+^ and Co^3+^ in the nanoparticles contribute to the adsorption of the compound on the surface of low-carbon steel by electrostatic interaction and the formation of bridges that bind the Fe^2+^-Cl^−^-CoO/Co_3_O_4_ NPs on the surface (Figure 5). Likewise, the presence of the free electron pair of donor O atom and the vacant d-orbits of the iron atoms on the surface, form coordinate bonds such as Fe-O-Co that create a layer that protects the surface from continuing corrosion [22,25].

The presence of the inhibitor ions (Co^2+^, Co^3+^) and the O atom enhances the adsorption of the inhibitor on the low-carbon steel surface and forms a protective film, and the decrease in the solubility of cobalt increases the efficiency of the inhibitor.

### 3.3. Determination of Thermodynamic Parameters and Adsorption Isotherm

The nanoparticles were adsorbed onto the low-carbon steel surface at the metal/solution interface. Since there are O atoms in the NPs of cobalt oxides, each particle can be considered to have a free electron pair-rich site(s), which can react with iron ions on the surface of the steel. Thus, the nanoparticles are adsorbed on the surface with high efficiency [26].

**Figure 6:**
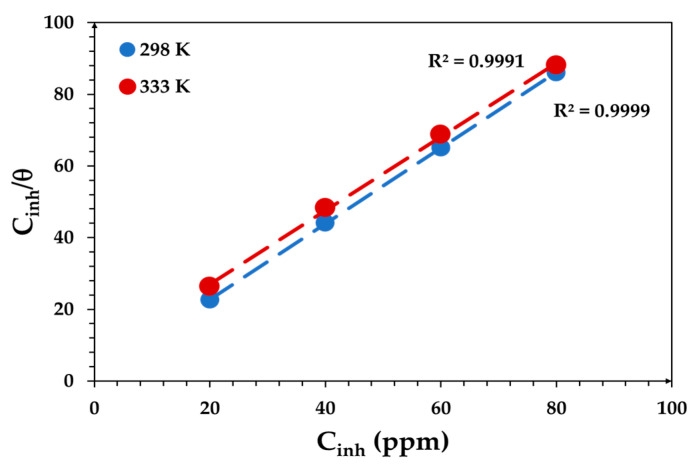
Langmuir adsorption isotherm plot of low-carbon steel corrosion in HCl with different concentrations of CoO/Co_3_O_4_ NPs at 298 and 333 K.

To describe the adsorption mechanism and find the important parameters that describe the type of interactions occurring between the metal and inhibitor, the Langmuir isotherm model was applied at 298 and 333 K, which is expressed as follows [4,6]:

(7) $$\mathrm{Langmuir}:\;\frac{C_{\mathrm{inh}}}{\theta }=\frac{1}{K_{\mathrm{ads}}}+C_{\mathrm{inh}}\;\;$$

where *C*_inh_ is the inhibitor concentration and *θ* is the surface coverage obtained from the EIS measurement, it is calculated using the following equation:

(8) $$\theta =\frac{i_{\mathrm{corr}}-i_{\mathrm{corr}\left(\mathrm{inh}\right)}}{i_{\mathrm{corr}}}$$

where *K*_ads_ is the adsorption equilibrium constant. *C*_inh_/*θ* vs. *C*_inh_ plotted as shown in Figure 6. A linear relationship and a correlation coefficient close to 1 (R^2^ > 0.999) was obtained (Figure 6), which means that the adsorption of CoO/Co_3_O_4_ NPs onto the low-carbon steel surface follows the Langmuir adsorption isotherm [27].

The K_ads_ values obtained from the Langmuir isotherm were used to calculate the values of adsorption standard Gibbs free energy (Δ*G*°_ads_).

The following equations were used to calculate the Gibb’s free energy (Δ*G*°_ads_), adsorption enthalpy (Δ*H*°_ads_), and adsorption entropy (Δ*S°*_ads_) of this system [26,28]:

(9) $$G{{}^{\circ}}_{\mathrm{ads}}=-RT\ln\left(1\times 10^{6}K_{\mathrm{ads}}\right)\;$$

(10) $$\frac{G{{}^{\circ}}_{\mathrm{ads}}\;}{T_{2}}-\frac{G{{}^{\circ}}_{\mathrm{ads}}\;}{T_{1}}=H{{}^{\circ}}_{\mathrm{ads}}\left(\frac{1}{T_{2}}-\frac{1}{T_{1}}\;\right)$$

(11) $$S{{}^{\circ}}_{\mathrm{ads}}=\frac{H{{}^{\circ}}_{\mathrm{ads}}-G{{}^{\circ}}_{\mathrm{ads}}\;}{T}\;$$

where *R* is the universal gas constant, *T* (*K*) is the absolute temperature and 1 × 10^6^ ppm is the concentration of adsorbed water molecules.

In general, values of Δ*G*°_ads_ less than −20 kJ/mol correspond to electrostatic reactions between the inhibitor and the low-carbon steel surface (physisorption). Similarly, values that are lower than −40 kJ/mol involve sharing the charge or transfer from inhibitor to the low-carbon steel surface to form a coordinate bond (chemisorption) [4,5,6].

The thermodynamic parameters listed in Table 1, which shows that the value of Δ*G*°_ads_ is −32.9 kJ/mol at 298 K, and −33.1 at 333 K, the interaction of the metal surface with the inhibitors is attributed to physical attraction, thus introducing a physisorption adsorption process. In addition to chemical bonding, as CoO/Co_3_O_4_ NPs contains many O atoms that have single-pair electrons and π-conjugate electrons, the coordinated interaction can be formed via charge sharing and transfer, thus introducing a chemisorption adsorption process. Therefore, CoO/Co_3_O_4_ NPs adsorption on the surface of low-carbon steel surface is a spontaneous physicochemical adsorption process [29,30].

The negative value of Δ*H*°_ads_ indicates that the adsorption is exothermic, and the positive value of Δ*S°*_ads_ indicates an increase in randomness due to the competition of nanoparticles ions at the active sites on the low-carbon steel surface [31].

The oxides nanoparticles tend to agglomerate due to strong interparticle magnetic attractions, van der Waals force, and high surface energy [32]. As small nanoparticles rapidly fuse to reach thermodynamic stability and transform into large-sized particles. Thus, the agglomeration process is thermodynamically controlled, and this is evident from the values we obtained in Table 1, that the values of *ΔG°*_ads_ were almost constant for both temperatures (298, 333 K) [33], as a result of the thermodynamic stability of the protective layer formation from inhibitor particles agglomerated on the surface of low-carbon steel.

### 3.4. Electrochemical Measurements

Electrochemical methods were used to study the concentration effect of the inhibitor on low-carbon steel corrosion in 1 M HCl at 298 and 333 K.

#### 3.4.1. The Electrochemical Impedance Technique

**Figure 7:**
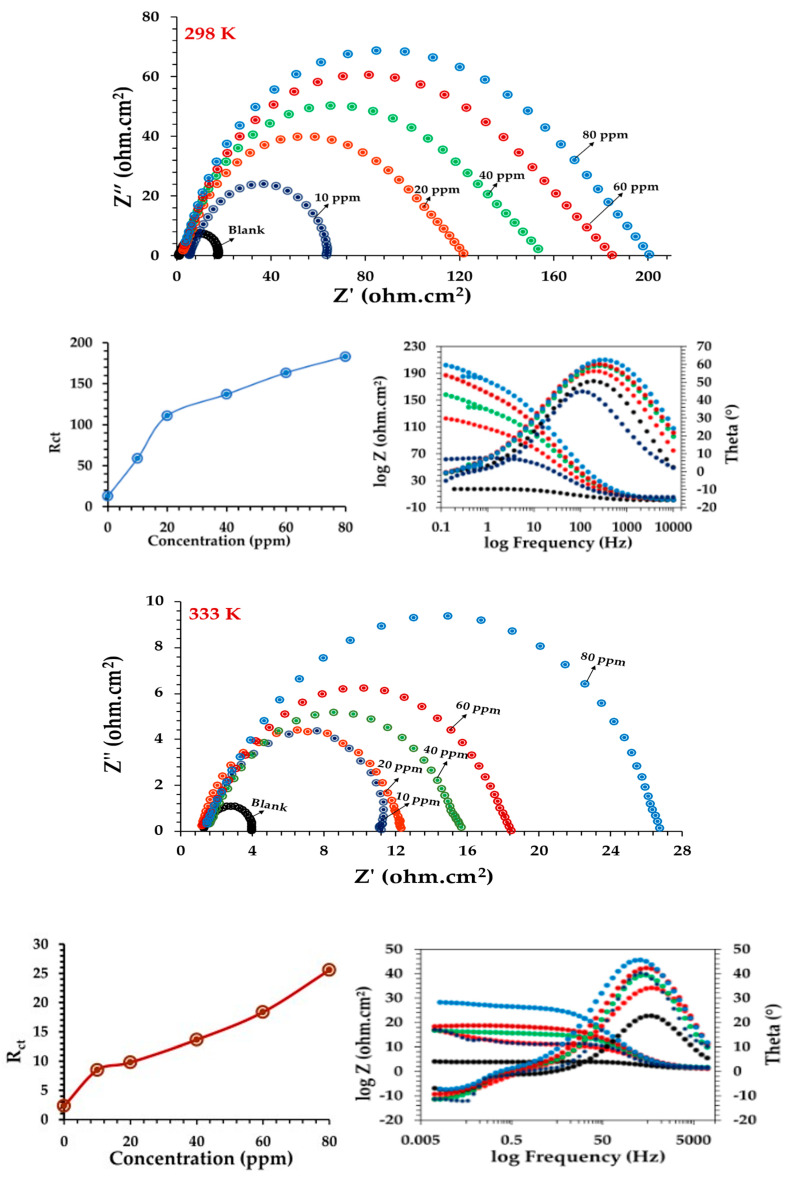
EIS diagrams of low-carbon steel corrosion in 1 M HCl in the absence and presence of CoO/Co_3_O_4_ NPs at 298 and 333 K after immersion for 30 min.

**Figure 8:**
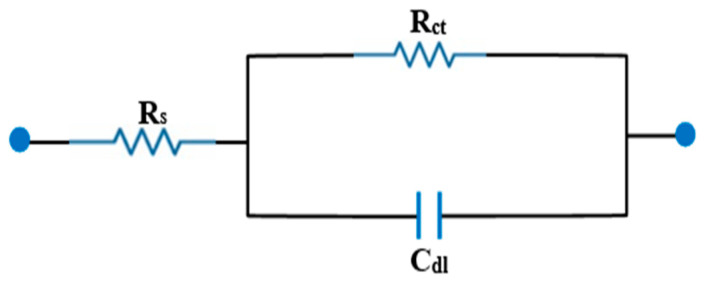
Equivalent circuit diagram.

By applying the EIS technique to determine the low-carbon steel’s behavior in the absence and presence of an inhibitor (Figure 7) and by comparing the Nyquist diagrams of the low-carbon steel in HCl in the absence and presence of CoO/Co_3_O_4_ NPs, it was observed that all the curves followed the same pattern; in this case, the results show a semicircle. The bode and phase angle diagrams showed increased area under the curves in the presence of the inhibitor compared to a blank solution. From the results in Table 2, it noted that the capacity of the double layer decreases and the resistance of the charge transfer increases with increasing concentration of inhibitor, and the inhibition efficiency increased from 59 to 93%. It is noted from Figure 7 that the charge transfer resistance decreases as the temperature increases to 333 K at the same concentration of CoO/Co_3_O_4_ NPs. Moreover, the inhibition efficiency decreased for all the inhibitor concentrations with increased the temperature from 298 to 333 K. The equivalent circuit of the results obtained from the electrochemical impedance measurements shown in Figure 8.

The results obtained from the EIS technique explained as follows. In the case of the blank solution, the presence of H^+^_ads_ and Cl^−^ ions in addition to the heterogeneity of the surface causes increased *C_*dl*_* and decreased *R_*ct*_* at the interface of the low-carbon steel surface/HCl solution [17,18]. In the case of the inhibitor, *R_*ct*_* increases and *C_dl_* decreases when adding CoO/Co_3_O_4_ NPs; additionally, as its concentration increases, the inhibitor particles compete with H_2_O, Cl^−^, and H^+^_ads_ ions for bonding with the Fe^2+^ ions on the low-carbon steel surface. The strong layer formed with the [Fe-2Cl-CoO/Co_3_O_4_], [Fe-O-CoO/Co_3_O_4_] and other complex are difficult to dissolve in an HCl solution [34,35]. Thus, this layer works to cover the exposed area of the electrode surface in the corrosion medium.

Therefore, the corrosion inhibition process is controlled by the adsorption process and not by the diffusion of the nanoparticles on the surface [36,37]. This indicates that the inhibitor acts as a mixed corrosion inhibitor that impedes both the anodic and cathodic reactions by replacing H_2_O molecules with nanoparticles at the interface of the low-carbon steel surface/HCl solution while also adsorbing these nanoparticles to create a protective film on the low-carbon steel surface that protects it from further corrosion [38,39].

#### 3.4.2. The Potentiodynamic Polarization Technique

**Figure 9:**
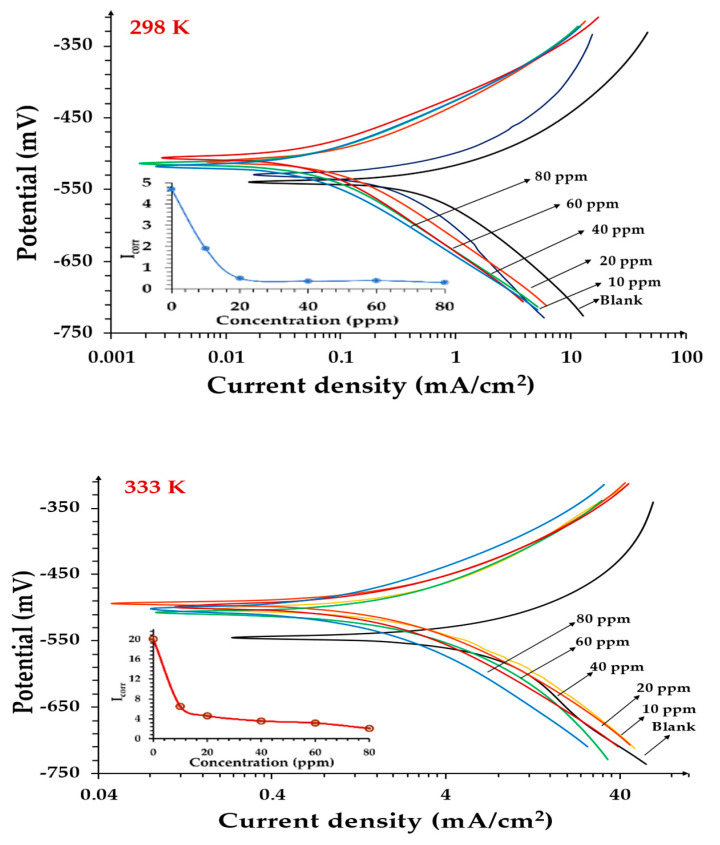
PDP plots of low-carbon steel corrosion in 1 M HCl in the absence and presence of CoO/Co_3_O_4_ NPs at 298 and 333 K.

PDP curves are plotted for the corrosion of low-carbon steels in the absence and presence of different concentrations of CoO/Co_3_O_4_ NPs (Figure 9) at 298 and 333 K, and the curves show a clear displacement in the anode and cathode Tafel slopes with increasing inhibitor concentration. Additionally, the mechanism of the anode and cathode interaction changed, meaning that the inhibitor inhibits both reactions. Therefore, the inhibition efficiency changed from 57 to 94%, when the concentration of inhibitor increased from 10 to 80 ppm, and the inhibition efficiency reached a maximum of 94% at 298 K and 89% at 333 K. The values of the corrosion current density (*I_*corr*_*), cathodic (*β*_c_), and anodic (*β*_a_) Tafel slopes and inhibition efficiency (*IE_*inh*_*) were obtained by extrapolating the polarization curve to the *IE_*corr*_* corrosion voltage and are listed in Table 3.

As shown in Table 3, it appears that the corrosion current decreases with an increasing concentration of inhibitor, and the corrosion potential shifts to less negative values. That is, the corrosion potential shifts toward the anode, which means that the presence of the inhibitor inhibits the anodic reaction more than the cathodic reaction [40]. In other words, CoO/Co_3_O_4_ NPs act as a mixed type of inhibitor, with the predominant anodic effect. The interaction occurs between the electrons in the inhibitor and the unoccupied d orbital of iron atoms, as well as the donor-acceptor interactions between the lone electron pairs of the O atoms and the vacant d orbital of the steel surface atoms. Where O atoms have single-pair electrons and π-conjugate electrons. In addition, the maximum transformation of *E*_corr_ is less than 85 mV, which means that CoO/Co_3_O_4_ NPs are a mixed-type corrosion inhibitor [41,42]. We also note that the inhibition efficiency of the anodic and cathodic reaction increases with an increasing concentration of inhibitor from 10 to 40 ppm, and after concentration 40 ppm, the increase in efficiency is not noticeable. The increase in the efficiency of the inhibitor with the increase in concentration is due to an increase in the thickness of the layer formed on the surface of the low-carbon steel, which serves to protect the low-carbon steel from continuous corrosion and becomes almost stable at concentrations higher than 40 ppm.

The results of the PDP agreed with those obtained from EIS.

### 3.5. Scanning Electron Microscopy (SEM), Energy-Dispersive X-ray Spectroscopy (EDS) and X-ray Electron Spectroscopy

**Figure 10:**
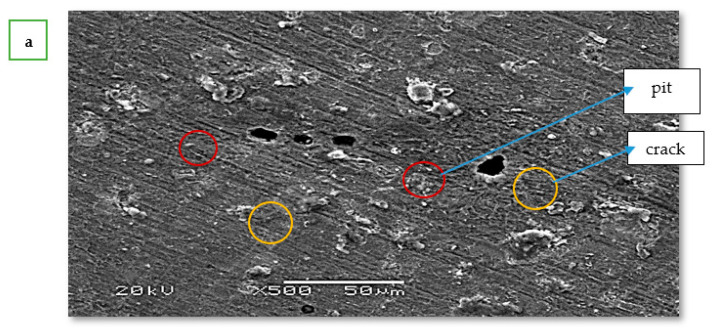

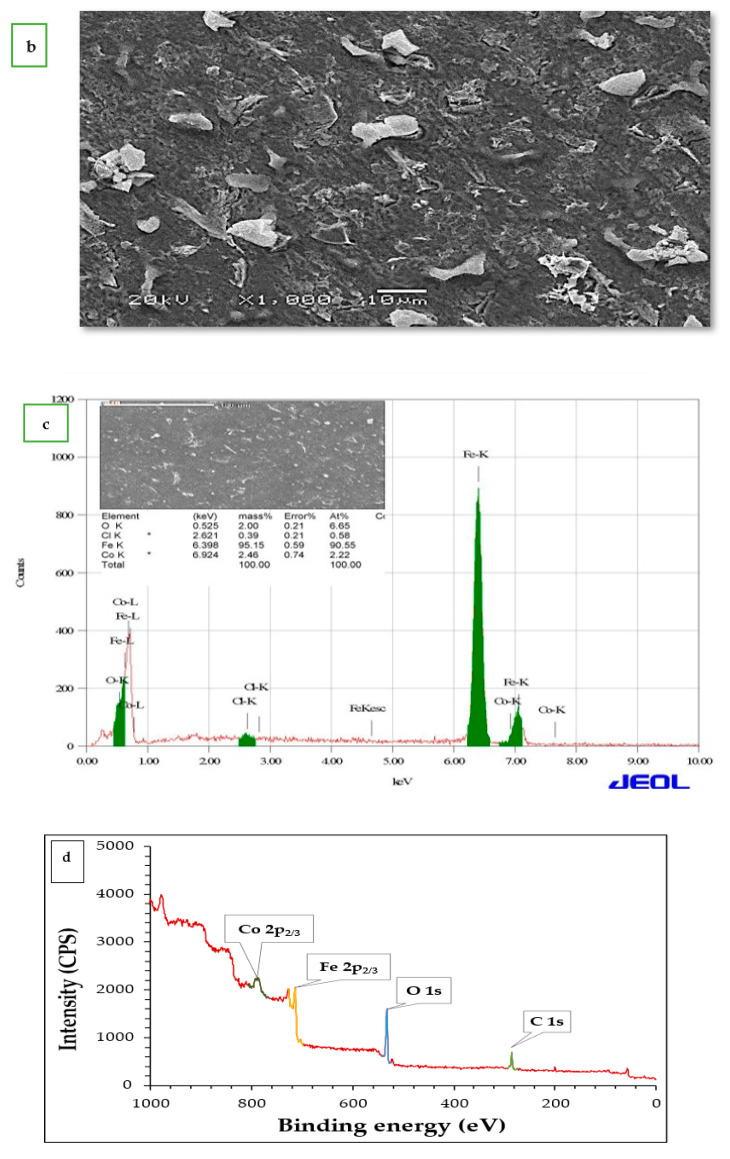
SEM image of low-carbon steel corrosion in 1 M HCl in the blank (**a**), SEM image (**b**), EDS spectra (**c**), and XPS (**d**) of low-carbon steel corrosion in 1 M HCl in the absence and presence of 40 ppm CoO/Co_3_O_4_ NPs.

The surface morphology of low-carbon steel was studied by scanning electron microscopy and energy dispersive spectroscopy after immersion for 30 h in 1 M HCl solution in the absence and presence of 40 ppm of CoO/Co_3_O_4_ NPs. Figure 10a shows cracks, pits, and scratches, as well as corrosion deposits on the low-carbon steel surface. From Figure 10b, it can be seen that deposits of CoO/Co_3_O_4_ NPs form on the surface, and thus a protective layer is formed on the surface of low-carbon steel. As shown in Figure 10c, the presence of O, Co, and C atoms signals indicate the adsorption of the inhibitor on the surface of low-carbon steel, and its association with the active sites on the metal surface [43]. Furthermore, the multiplicity of the Co atom signals and the decrease in the signals of the Cl atom with the high signal of the Fe atom, indicate the high efficiency of the inhibitor in reducing the corrosion rate and protecting the low-carbon steel surface [22,44].

XPS was performed on the surface of low-carbon steel after being immersed in a 1 M HCl containing 40 ppm of CoO/Co_3_O_4_ NPs. The high-resolution C 1s, O 1s, Fe 2p, and Co 2p signals, O 1s dissociation spectrum was obtained in Figure 10d, which can be attributed to the presence of Fe^3+^ bound oxides. A spectrum of Fe 2p atom was also observed (Figure 10d) attributed to the presence of ferric ion complexes such as Fe_2_O_3_ and/or Fe_3_O_4_ [45]. A spectrum of Co 2p atom was also observed (Figure 10d) attributed to the presence of cobalt ion complexes such as CoO and/or Co_3_O_4_, which form complexes with iron. The XPS results confirm the adsorption of CoO/Co_3_O_4_ NPs on the low-carbon steel surface.

## 4. Conclusions

The efficacy of CoO/Co_3_O_4_ nanoparticles to prevent corrosion of carbon steel in 1 M HCl solution was studied. We will focus on inhibitor concentrations from 10 to 80 ppm for more details in order to find the limit of their effectiveness. When the Langmuir adsorption isotherm model was applied, it was found that the linear relationship was close to unity (R^2^ = 0.999), meaning that the inhibitor adsorption process on the low-carbon steel surface followed the Langmuir isotherm. The values of the thermodynamic parameters (ΔG°_ads_, ΔH°_ads_, and ΔS°_ads_) showed that CoO/Co_3_O_4_ NPs were spontaneously adsorbed onto the low-carbon steel surface by mixed adsorption (physical and chemical adsorption); additionally, the process was exothermic, and the positive value of entropy was the result of competition between the inhibitor particles at the active sites on the low-carbon steel surface. Electrochemical measurements (EIS and PDP) showed that the inhibition efficiency of the CoO/Co_3_O_4_ NPs inhibitor increases with increasing concentration from 10 to 40 ppm. The increase in the efficiency of the inhibitor with increasing concentration is due to the increased thickness of the layer formed on the surface of the low carbon steel, which serves to protect the low carbon steel from continuous corrosion and become almost stable at concentrations above 40 ppm. The CoO/Co_3_O_4_ NPs had a high efficiency of 93% at a concentration of 80 ppm and a temperature of 298 K, while the efficiency was 90% at 333 K. The inhibitor acted as a mixed inhibitor that affected both the anodic and cathodic reactions at the interface of the low-carbon steel/HCl solution. Furthermore, the results obtained from SEM and EDS confirmed that the presence of adsorbed deposits on the surface with the appearance of signals for O, Co, and C atoms indicates the adsorption of the inhibitor onto the low-carbon steel surface and its effectiveness in protecting the low-carbon steel surface.

## Figures and Tables

**Table 1:** Thermodynamic parameters of low-carbon steel corrosion in HCl with different concentrations of CoO/Co_3_O_4_ NPs at 298 and 333 K.

Temperature (K)	∆*G*°_ads_ (kJ/mol)	∆*H*°_ads_ (kJ/mol)	∆*S*°_ads_ (kJ/mol K)
298	−32.9	−31.2	5.8
333	−33.1		

**Table 2:** EIS parameters of low-carbon steel corrosion in 1 M HCl in the absence and presence of CoO/Co_3_O_4_ NPs at 298 and 333 K.

Concentration (ppm)		298 K			333 K			
	*R_*sol*_* (ohms∙cm^2^)	*C_*dl*_* (µF)	*R_*ct*_* (ohms∙cm^2^)	*E_*inh*_* (%)	*R_*sol*_* (ohms∙cm^2^)	*C_*dl*_* (µF)	*R_*ct*_* (ohms∙cm^2^)	*E_*inh*_* (%)
Blank	1.94	363	13	0	1.60	791	2.3	0
10	1.83	253	59	59	1.68	450	8.46	73
20	1.70	198	111	88	1.71	445	9.8	76
40	1.56	146	137	91	1.78	337.6	13.6	83
60	1.43	133	163	92	1.81	330.8	16.4	86
80	0.034	96	183	93	1.45	319.6	25.6	91

**Table 3:** PDP parameters of low-carbon steel corrosion in 1 M HCl in the absence and presence of CoO/Co_3_O_4_ NPs at 298 and 333 K.

Concentration (ppm)	298 K					333 K				
	*E*_corr_ (mV)	*β*_a_ (mV/dec)	*β*_c_ (mV/dec)	*I*_corr_ (mA/cm^2^)	*E*_inh_ (%)	*E*_corr_ (mV)	*β*_a_ (mV/dec)	*β*_c_ (mV/dec)	*I*_corr_ (mA/cm^2^)	*E*_inh_ (%)
Blank	−539	125	137	4.7	0	−545	217	202	20.0	0
10	−528	100	129	1.9	57	−504	142	198	5.6	72
20	−511	89	111	0.5	89	−493	129	157	4.6	77.1
40	−513	82	105	0.4	92	−506	127	197	3.6	82.3
60	−505	75	110	0.4	92	−499	120	153	3.2	84.3
80	−517	80	101	0.3	94	−501	118	159	2.1	89.8

## Data Availability

Not applicable.
